# Retraction: Epigenetic silenced miR‐125a‐5p could be self‐activated through targeting Suv39H1 in gastric cancer

**DOI:** 10.1111/jcmm.15847

**Published:** 2021-01-04

Epigenetic silenced miR‐125a‐5p could be self‐activated through targeting Suv39H1 in gastric cancer. Mingzhi Cai, Qiuxian Chen, Juntao Shen, Chenbing Lv and Lisheng Cai. J Cell Mol Med. 2018; 22: 4721‐4731.

The above article from Journal of Cellular and Molecular Medicine, published online on 17 August 2018 on Wiley Online Library (wileyonlinelibrary.com) and in Volume 22, pp. 4721‐4731, has been retracted by agreement between the authors, the journal Editor‐in‐Chief and John Wiley & Sons Ltd. The retraction has been agreed because parts of the data and figures that the authors presented in the paper are flawed. The conclusions drawn from the data and figures presented could not be considered as reliable.

